# Keeping up with the Wangs: individual and contextual influences on mental wellbeing and depressive symptoms in China

**DOI:** 10.1186/s12889-022-12869-8

**Published:** 2022-03-29

**Authors:** R. Adele H. Wang, Claire M. A. Haworth, Qiang Ren

**Affiliations:** 1 School of Economics, University of Bristol, Bristol, UK; 2 School of Psychological Science, University of Bristol, Bristol, UK; 3 Department of Population Health Science, Bristol Medical School, University of Bristol, Bristol, UK; 4 NIHR Biomedical Research Centre at the University Hospitals Bristol NHS Foundation Trust and the University of Bristol, Bristol, UK; 5 Centre for Social Research, Guanghua School of Management, Peking University, Beijing, China

**Keywords:** China, Wellbeing, Contextual, Depression, Happiness, Life satisfaction

## Abstract

**Background:**

In recent decades, China has experienced dramatic changes to its social and economic environment, which has affected the distribution of wellbeing across its citizens. While several studies have investigated individual level predictors of wellbeing in the Chinese population, less research has been done looking at contextual effects. This cross-sectional study looks at the individual and contextual effects of (regional) education, unemployment and marriage (rate) on individual happiness, life satisfaction and depressive symptomatology.

**Methods:**

Data were collected from over 29,000 individuals (aged 18 to 110, 51.91% female) in the China Family Panel Studies, and merged with county level census data obtained from the 2010 China Population Census and Statistical Yearbook. To explore contextual effects, we used multilevel models accounting for the hierarchical structure of the data.

**Results:**

We found that a one-year increase in education was associated with a 0.17% increase in happiness and a 0.16% decrease in depressive symptoms. Unemployed men were 1% less happy, 1% less satisfied with life and reported 0.84% more depressive symptoms than employed men while minimal effects were seen for women. Single, divorced and widowed individuals had worse outcomes than married individuals (ranging from 2.96 to 21% differences). We found interaction effects for education and employment. Less educated individuals had greater happiness and less depressive symptoms in counties with higher average education compared to counterparts in less educated counties. In contrast, more educated individuals were less satisfied with life in more educated counties, an effect that is possibly due to social comparison. Employed individuals had lower life satisfaction in areas of high unemployment, while levels were constant for the unemployed. A 1% increase in county marriage rate was associated with 0.33 and 0.24% increases in happiness and life satisfaction respectively, with no interactions. We speculate that this effect could be due to greater social cohesion in the neighbourhood.

**Conclusions:**

Our results show that policies designed to improve employment and marriage rates will be beneficial for all, while interventions to encourage positive social comparison strategies may help to offset the negative effects of increasing neighbourhood average education on the highly educated.

**Supplementary Information:**

The online version contains supplementary material available at 10.1186/s12889-022-12869-8.

## Background

Over the past decade, interest in wellbeing has expanded exponentially across the world. Most countries now realise the value of using wellbeing as a policy goal and a measure of social progress. Mental wellbeing, in particular, is important, as it leads to positive outcomes for the individual in other domains of life such as relationships, work productivity and physical health [1], which in turn impacts a whole country’s stability and economic prospects. Much of the research investigating mental wellbeing has been based on Western samples but these findings cannot be generalised to other countries [2, 3]. China is an especially interesting country in which to study mental wellbeing as it has experienced dramatic changes to its social and economic environment. These changes have not been uniform across the country which has resulted in China shifting from being one of the most egalitarian countries in terms of life satisfaction to one of the least [4]. Compared to the past decades, there is now a much higher discrepancy in percentage of people with high life satisfaction amongst those with the highest and the lowest incomes and educational qualifications. Among those in the highest income and better educated group, the percentage of people with high life satisfaction has increased but in contrast, the lowest income group has experienced substantial declines in life satisfaction over China’s transition period [4].

There is an abundance of research that has investigated the influence of individual level socio-demographic and socio-economic characteristics on mental wellbeing, showing the benefits of belonging to a higher socioeconomic class: having a higher income (up to a certain value), having more education, being employed and being married [5, 6]. These positive effects have also been shown in China [7–11]. However, people do not live in isolation and less attention has been paid to the influence of contextual level characteristics on mental wellbeing in China. This study aims to contribute to this relatively unexplored field.

In this study, we conceptualise mental wellbeing as life satisfaction and happiness. Life satisfaction is the most pervasive measure of wellbeing, both within research and policy. It requires a cognitive appraisal of one’s life [12]. Happiness captures hedonic wellbeing, which focuses on pleasure attainment and pain avoidance [13]. We also use a measure of depressive symptomology. Mental wellbeing and depression are two constructs that are correlated [14], but have genetic and environmental specificities which suggest the two to be aetiologically independent [15, 16]. We will now give an overview of the literature on contextual effects on mental wellbeing and depressive symptoms/depression. Unless specified, the research reviewed is based on Western samples.

### Neighbourhood contextual influences

Studies examining neighbourhood contextual effects often use a composite measure of neighbourhood deprivation which takes into consideration aspects such as number of people living in poverty, ethnic heterogeneity and residential stability. Living in deprived neighbourhoods is associated with lower levels of life satisfaction and higher levels of depressive symptoms/depression incidence [17–23]. However the literature is mixed as other studies have found no association [18, 24, 25]. These inconsistent findings are typically due to heterogeneous measurements of neighbourhood characteristics, individual level control variables and outcomes.

Some studies have honed-in on specific aspects of the socioeconomic and socio-demographic contextual environment. Higher neighbourhood income and local employment levels have been independently shown to be associated with decreased risk of depression [26, 27] and higher individual wellbeing [6, 28–31]. One major limitation of these studies is that they often only examine a single neighbourhood factor, even though neighbourhood factors are highly intercorrelated. In our current study we investigate associations while adjusting for other relevant individual and neighbourhood factors.

There are several explanations as to why the inhabitants of richer neighbourhoods may have better outcomes. Advantaged neighbourhoods have the finances to provide services and amenities that can boost the mental health of its inhabitants. Socioeconomically advantaged neighbourhoods are high in social capital and low in social disorder, which bolsters public trust and availability of social support for individuals [19]. Individuals living in disadvantaged neighbourhoods may suffer in their mental health because they may feel sympathy for the many disadvantaged people around them, they may worry about the security of their own situation, or they may suffer from the repercussions of disadvantage in the entire region (e.g. crime, lower economy) [32].

In addition to main (absolute) effects, there are also interesting interactions (relative effects) between individual and contextual level factors on the outcomes of mental wellbeing and depression. Evidence shows that individuals are more satisfied with their lives when their own social status is higher than their neighbours [17]. Relative deprivation and relative income rank have also been shown to be associated with higher odds of depression [33]. When studies have looked at the interaction between individual unemployment and local unemployment, they find that the wellbeing of the unemployed tends to be higher in areas where unemployment rate is higher [28, 31, 34, 35]. Nikolaev [36] showed that relative regional education is negatively associated with happiness. These effects may be due to social comparison processes and the impact of social norms. Social comparison is when an individual compares his/her situation to the situation of others [37]. Most often, people compare themselves to others who are better off [38] and this activates a specific feeling of “relative deprivation” [36, 39]. This occurs when individuals do not feel that they meet the conditions of the social norm and so do not have their physical or psychological needs satisfied [40], this in turn lowering wellbeing [41]. Disadvantaged individuals who are living in more disadvantaged neighbourhoods may feel less stigma and externalise the reasons for their own situation, so their wellbeing tends to be better [28].

### Contextual effects in China

While evidence concerning contextual effects is abundant in the Western context, such findings cannot be directly translated to the situation in China. Cultural differences may play an important role in differentiating the effect of contextual factors in Eastern and Western cultures. Culture shapes how individuals judge people around them [42] and so it is very likely that culture will also shape how individuals judge themselves as they compare themselves with others. Studies investigating the contextual influences on wellbeing and mental health in China is sparse and predominately on income, showing positive effects of neighbourhood economics status, with relative income positively associated as well [10, 43, 44]. In addition to income, factors such as education, unemployment and relationship status are equally important to study in China. Education has been shown to be positively associated with subjective wellbeing in both rural and urban regions [8, 10, 11] while negative (and sometimes null) associations have been found with depression and mood disorders [9]. Unemployment is associated with lower wellbeing and increased likelihood of depression and mood disorders [7, 9]. Married individuals in China tend to be happier than those who are unmarried, with those who are widowed or divorced often suffering the most, being less happy, more depressed or having more symptoms of mood disorders [7, 9–11]. No studies have looked at the contextual effects of education, employment and relationship status on wellbeing and mental health in China, and such effects may be even bigger than the individual effects found, which may have policy implications.

This current study has two aims:

1. To explore individual level and contextual level effects of education, employment and relationship status on individual mental wellbeing and depressive symptomatology in China.
2. To see if the contextual effects are similar for individuals at both the higher and lower ends of education level, and for those of different employment and relationship statuses.

We used data from over 29,000 individuals spanning 18 to 110 years of age taken from the China Family Panel Studies (CFPS), a nearly nationwide social survey [45]. A common operationalisation of neighbourhoods is to use census tracts, which are geographical regions defined for the purpose of the census [46, 47]. Similarly in other countries such as the UK, Canada, Germany and the Netherlands, administratively defined areas such as electoral wards, regional unitary authorities, postcode areas and boroughs are commonly used to define neighbourhoods [47, 48]. In the current study we use county-level data, which is the lowest geographical subdivision available from the 2010 China Population Census and Statistical Yearbook. This is comparable with regions as used in Western studies. We explore effects on the entire 18 to 110 years age range, and we also look at individuals of working age, between 18 and 59, who will have different priorities especially in relation to their own employment status. Furthermore we split our analysis by sex, and urban-rural dwellers, given that significant differences have been found before with respect to our outcomes [7, 10].

## Methods

### Sample

This study used data from the 2010 wave of the large scale, nearly nationally representative China Family Panel Studies (CFPS). The CFPS is conducted by the Institute of Social Science Survey (ISSS), Peking University, and was designed to examine a range of topics including education, employment, family dynamics, health and child development [45]. All individuals from 14,798 households from 25 provinces in China were selected through a multistage probability sample procedure. Participants in CFPS represent geographical regions of China that contain 95% of the Chinese population in mainland China. CFPS samples covered 25 provinces in Mainland China, in which Tibet, Qinghai, Xinjiang, Ningxia, Inner Mongolia, and Hainan provinces were excluded from the sample to reduce costs. The exclusion of sampling from these provinces means that we cannot say that the sample is nationally representative but since the population within these excluded provinces make up only 5% of the population of China, estimates are unlikely to be affected. Questionnaire data were collected by interviewers through home visits. For better control of the implementation costs and time, CFPS employed local interviewers mainly from the sampled neighbourhood communities in the baseline survey. Each interviewer was in charge of two neighbourhood communities, and in big cities, 2–5 additional interviewers were required. Computer Assisted Personal Interviewing (CAPI) technology was used in CFPS. Participants were asked the questions out loud by the interviewers face to face. The interviewer presents the informed consent form to the interviewee, which must be read out if necessary, and the interview can only start after the interviewee signs and confirms it.

Corresponding with the legal age of marriage, females under 18 and males under 20 were excluded from the analysis. Also excluded were migrants, which we classified as those who both held a different household registration (“*hukou*”) to their current place of residence and also had not been living in the current area at the age of 3 or 12. Migration is a significant social phenomenon in China and migrants have been shown to have distinctly different demographic profiles while being allowed limited access to public services in their destination cities [49] – they were thus excluded from the current analyses to prevent additional confounding and bias in our model. In total, 3793 individuals were excluded and 29,807 were used in our main analyses though different individuals had different missing observations for our predictor, outcome and control measures. Given the minimal observation missingness (0–6%), we conducted complete case analyses. The mean age of these individuals was 47.18 (SD = 15.52, range = 18 to 110) and 51.94% were female.

Contextual data were obtained from the 2010 China Population Census and Statistical Yearbook., with county level data as the lowest geographical subdivision. While policies for government spending and social services are made at the level of the province, the policies are carried out based on financial availability at the county level. There are 2861 county units in Mainland China ranging from population sizes of 2215 to 5,044,430. The CFPS gathered information on 641 county units, with populations ranging from 44,867 to 5,044,430 (mean = 680,526, SD = 807,949). In our sample, the number of individuals in each county ranged from 33 to 347 (mean = 185, SD = 57).

### Measures

Details of the measures used are provided in Table 1, including descriptions, sample items and items ranges where applicable.

**Table 1:** Description of outcome, predictor and control variables used. (Page 7, Line 174)

Measure		Description	Example item	Item range/ categories	Mean (SD)/ Frequency
Outcomes^a^	Happiness	Single item	“How happy are you?”	1 (low happiness) to 5 (high happiness)	3.82 (1.02)
	Life satisfaction	Single item	“How satisfied are you with your life as a whole these days?”	1 (low life satisfaction) to 5 (high satisfaction)	3.47 (1.04)
	Depressive symptomatology	Short form of the Centre for Epidemiologic Studies Depression Scale (CES-D) - sum composite of 6 items	“How often in the past month did you feel so depressed that nothing could cheer you up?”	6 to 30	12.12 (3.95)
Individual Level Predictors	Education level	Total number of years spent in education		1 to 22	6.77 (4.81)
	Employment status	Dichotomous variable of employed or unemployed		0 (employed) and 1 (unemployed)	49.59% unemployed
	Relationship status	A set of dummy variables, with being married as the comparative baseline and a set of dummy variables representing (1) single, (2) cohabiting, (3) divorced, and (4) widowed			83.03% married (8.91% single, 0.22% cohabiting, 1.38% divorced, 6.46% widowed)
County Level Predictors	County average education	Average education level in county			8.94 (1.38)
	County unemployment rate	Proportion unemployed in county		0 to 1	0.33 (0.10)
	County marriage rate	Proportion married in county		0 to 1	0.71 (0.04)
Control Variables	Age			18 to 110	47.18 (15.52)
	Gender			0 (male) and 1 (female)	51.94% female
	Ethnicity	Han-Chinese or not Han-Chinese		0 (Han) and 1 (non-Han)	8.42% non-Han
	Urban	Living in urban or rural area, categorised by 2010 census^b^		0 (rural) and 1 (urban)	44.22% urban
	Health	Single item	“How would you rate your health status?”	1 to 5	1.86 (1.04)
	Log income per capita	Net household income per capita (logarithm transformed)			9152.17^d^ (15,094.24)
	Log county GDP per capita	County GDP per capita (from census data)			40,458.11^d^ (52,841.14)
	Log asset per capita	Net family asset per capita (logarithm transformed)			83,174.58^e^ (211,855.63)
	Log county asset per capita	County average family asset per capita (logarithm transformed)^a^			83,912.10^c^ (116,575.67)

^a^ Happiness and life-satisfaction are single item questions, which are widely used both in English and Chinese literature. Short form of the Centre for Epidemiologic Studies Depression Scale (CES-D) was adapted in this study, which has been validated for studies of Chinese adults [50–52]

^b^”Urbanness” includes urban areas and towns. The urban area refers to the neighborhood committees and other areas that are connected to the actual construction of the municipal districts and cities without districts. The town district refers to the residents’ committee and other areas connected to the actual construction of the county government and other towns outside the urban area. “ruralness” refers to the area outside the towns delineated by “urbanness” regulations

^c^ Calculated using a cell-average approach [28, 36] - for every individual, their corresponding county average family asset variable is calculated by an average of the family asset of every other individual in the same county

^d^Income per capita in RMB

^e^Assets per capita RMB

Our outcome variables are happiness, life satisfaction and depressive symptomatology. Happiness was measured using a self-reported single item: “How happy are you?” and life satisfaction was measured using a self-reported single item: “How satisfied are you with your life as a whole these days?”. Participants were asked to rate their happiness and life satisfaction on a 5-point scale ranging from 1 (low happiness/life satisfaction) to 5 (high happiness/life satisfaction). Depressive symptomatology was a sum composite of 6 items from the short form of the Centre for Epidemiologic Studies Depression Scale (CES-D). For each item, respondents were asked how often they felt this way during the past month: almost every day, two or three times a week, two or three times a month, once a month, or never. Each item, such as “How often in the past month did you feel so depressed that nothing could cheer you up?”, were rated on a 5-point scale (ranging from 1 “never” to 5 “almost every day”) and the overall depression scored ranged from 6 to 30 where a higher score meant greater depressive symptomatology. The 6 items have a Cronbach’s alpha of 0.85.

### Statistical analysis

A multilevel analytical approach is needed to explore contextual effects [21, 53]. This accounts for the hierarchical structure of the data. In the current study, we build multilevel models with individuals nested within families, then nested within county. For each outcome, we built two models – the first to examine the main effects of education, employment and relationship status (individual and contextual), and the second to examine if individual level and contextual level factors interact, including control variables in each. Altogether, for each of our 3 outcomes, we statistically tested 6 individual level predictors (education, employment status and 4 relationship dummy variables), 3 contextual level predictors (county average education, employment rate and marriage rate) and 9 interaction effects (between individual level and county level predictors of education, employment and relationship). We used the Benjamini-Hochberg False Discovery Rate (FDR) Procedure to control for multiple testing of these effects of interest to give us an indication of the strongest, most robust effects. We also repeated this analysis on individuals of working age. Furthermore we split our analysis by sex, and urban-rural dwellers.

## Results

Mean and standard deviation of our predictor and outcome variables are given in Table 1. Supplementary Tables 1 and 2 give the breakdown of employment status and marital status. Supplementary Table 3 presents the correlations between our predictors and outcomes of interest.

A multilevel model was used for our main analysis, with estimates of coefficients obtained through maximum likelihood. Table 2 show the effects of individual and county level education, employment status and relationship status on happiness, life satisfaction and depressive symptomatology respectively. Supplementary Table 4 shows the model for participants aged 18 to 59, which is the official age before retirement, with comparable results.

**Table 2:** Wellbeing and depressive symptoms predicted by individual and contextual level factors (Page 8, Line 204)

	Happiness		Life Satisfaction		Depressive symptoms	
	Estimate	SE	Estimate	SE	Estimate	SE
Intercept	0.78	0.46	0.41	0.46	12.97	1.67
Years in Education	8.40E-03***^a^	1.64E-03	1.21E-03	1.69E-03	−0.04***^a^	6.04E-03
Employment status	−6.75E-04	0.01	− 1.26E-04	0.01	0.03	0.05
*Relationship status dummy set*						
Single	−0.25***^a^	0.03	− 0.14***^a^	0.03	0.74***^a^	0.10
Cohabitation	−0.25*	0.12	−0.10	0.13	1.03*	0.46
Divorced	−0.59***^a^	0.05	−0.48***^a^	0.05	0.93***^a^	0.18
Widowed	−0.29***^a^	0.03	−0.19***^a^	0.03	1.05***^a^	0.10
County average education	0.02	0.03	3.78E-03	0.03	−0.09	0.10
County unemployment rate	0.17	0.25	−0.31	0.26	0.25	0.93
County marriage rate	0.02***^a^	4.24E-03	0.01**^a^	4.32E-03	−2.58E-02	1.55E-02
Age	−0.04***	2.48E-03	−0.02***	2.55E-03	0.01	9.15E-03
Age squared	4.49E-04***	2.47E-05	3.45E-04***	2.54E-05	−2.60E-04**	9.13E-05
Female	0.10***	0.01	0.10***	0.01	0.28***	0.04
Non Han	0.03	0.03	7.79E-03	0.03	−0.24*	0.12
Urban	0.02	0.02	−0.10***	0.02	4.04E-03	0.07
Health	−0.16***	5.95E-03	−0.15***	6.14E-03	1.27***	0.02
Log income per capita	0.11***	7.92E-03	0.14***	8.07E-03	−0.29***	0.03
Log asset per capita	0.18***	0.03	0.23***	0.03	−0.34***	0.10
Log county GDP per capita	0.05	0.03	−0.02	0.03	−0.22	0.12
Log county asset per capita	−0.11**	0.04	−0.09*	0.04	0.03	0.13
Observations	26,682		27,196		26,981	
N_families_	12,501		12,521		12,470	
N_counties_	158		158		158	
AIC	72,305.24		75,537.70		143,689.74	

Note. Multilevel model with individual and contextual education, employment status and relationship status predicting happiness, life satisfaction and depressive symptoms. Employment status was coded as 0 for being employed and 1 for being unemployed. Relationship status was added into the model as a set of dummy variables. The relationship dummy set’s reference state was being married. *p*-values were adjusted using the Benhamini-Hochberg False Discovery Rate Procedure to control for multiple testing of the effects of interest, thus we only indicate FDR significance for our predictors of interest – individual and contextual level of education, employment and marriage

*p < 0.05, **p < 0.01, ***p < 0.001, ^a^FDR

### Individual level correlates of wellbeing and depressive symptomatology

At the individual level, we find evidence for a positive association between education and happiness (*b* = 8.40E-03, SE = 1.64E-03, *p* < 0.001) and a negative association between education and depressive symptomatology (*b* = − 0.04, SE = 6.04E-03, *p* < 0.01). The significance of these findings is driven by the standard errors and we note that the point estimates are very small and of low ecological importance. Spending one more year in education results in a 0.17% increase in happiness, and 0.16% decrease in depressive symptomatology.

We found no association between unemployment and wellbeing or depressive symptomatology. Supplementary Table 5 shows results from models where unemployment was a set of dummy variables modelling the different reasons given for unemployment. The strongest effects in the table show that those who are unemployed because they have enough economic capability and hence have no need to work are happier (*b* = 0.21, SE = 0.06, *p* < 0.001) and more satisfied with life (*b* = 0.28, SE = 0.07, *p* < 0.001) than the employed. Those who are unemployed due to retirement are happier (*b* = 0.06, SE = 0.03, *p* < 0.05), more satisfied with life (*b* = 0.10, SE = 0.03, *p* < 0.001) and less depressed (*b* = − 0.82, SE = 0.10, *p* < 0.001) than the employed. Those who are too old and feeble to work are happier than the employed (*b* = 0.07, SE = 0.02, *p* < 0.01). Those who have no working capability due to disability and illness are less happy (*b* = − 0.10, SE = 0.04, *p* < 0.01) and have more depressive symptoms (*b* = 1.57, SE = 0.13, *p* < 0.001) than the employed. The unemployment variable in this model had a lot of missing data, with many unemployed individuals responding with “other” or not giving a reason at all.

Single individuals are 5% less happy (*b* = − 0.25, SE = 0.03, *p* < 0.001), 2.8% less satisfied with their life (*b* = − 0.14, SE = 0.03, *p* < 0.001) and 2.96% more depressed (*b* = 0.74, SE = 0.10, *p* = < 0.001) than married individuals. Divorced individuals are 11.8% less happy (*b* = − 0.59, SE = 0.05, *p* < 0.001), 9.6% less satisfied with their life (*b* = − 0.48, SE = 0.05, *p* < 0.001) and 3.72% more depressed (*b* = 0.93, SE = 0.18, *p* < 0.001) than married individuals. Widowed individuals are 5.8% less happy (*b* = − 0.29, SE = 0.03, *p* < 0.001), 3.8% less satisfied with their life (*b* = − 0.19, SE = 0.03, *p* < 0.001) and 21% more depressed (*b* = 1.05, SE = 0.10, *p* < 0.001) than married individuals. We found weak (greater standard error) evidence to suggest that cohabiting individuals were less happy (*b* = − 0.25, SE = 0.12, *p* < 0.05) and more depressed (*b* = 1.03, SE = 0.46, *p* < 0.05) than married individuals.

### Contextual level correlates of wellbeing and depressive symptomatology

At the contextual level, we did not find evidence to support an association between county average education level or county unemployment rate and individual wellbeing or depression. We did find evidence that county level marriage rate is positively associated with individual happiness (*b* = 0.02, SE = 4.24E-03, *p* < 0.001) and life satisfaction (*b* = 0.01, SE = 4.32E-03, *p* = < 0.01). This means that a 1% increase in the marriage rate within a county is associated with a 0.33% increase in individual happiness level and a 0.24% increase in individual life satisfaction level.

### Interaction effects between individual and contextual level factors

**Fig. 1:**
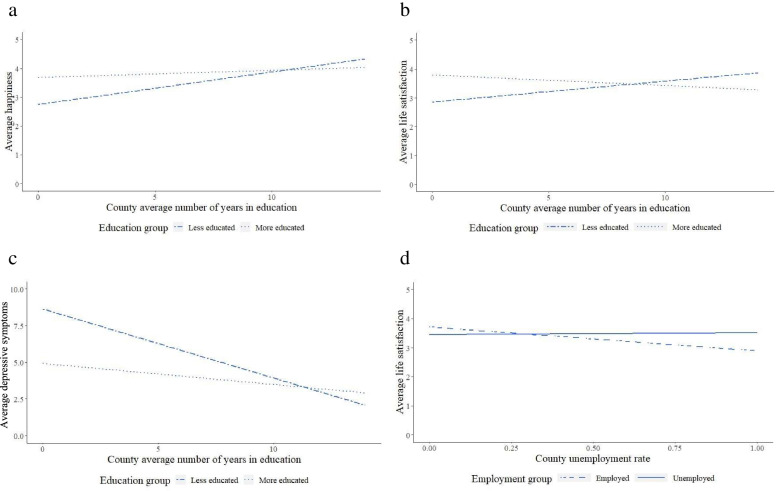
Interactions effects of individual and county level factors on wellbeing and depressive symptoms. Note. Line type refers to individual education level. **a** Interaction effect of individual and county education on happiness. Individuals were placed in the less educated group if their total number of years spent in education was less than the national average (8.95 years) and placed in the more educated group if they spent more years in education than the national average. Graphs shows that both high and low educated individuals are happier in more educated counties, though the effect is greater for less educated individuals. **b** Interaction effect of individual and county education on life satisfaction. Individuals were placed in the less educated group if their total number of years spent in education was less than the national average (8.95 years) and placed in the more educated group if they spent more years in education than the national average. Graphs shows that high and low educated individuals react differently to county education average. The more educated individuals are more satisfied with life in counties where average education is lower, while the opposite is true for less educated individuals. **c** Interaction effect of individual and county education on depressive symptoms. Individuals were placed in the less educated group if their total number of years spent in education was less than the national average (8.95 years) and placed in the more educated group if they spent more years in education than the national average. Graphs shows that both high and low educated individuals report fewer depressive symptoms in more educated counties, though the effect is greater for less educated individuals. **d** interaction effect of individual unemployment status and county unemployment rate on life satisfaction. This graph shows that the employed are less satisfied with life in counties where the unemployment rate is high, while there are minimal differences in life satisfaction for the unemployed

Table 3 shows the results of our models that tested for the existence of interaction effects between the individual and contextual levels factors on our outcomes. We found evidence for interaction effects between individual and county average education on all three outcomes. The interaction effect of education on happiness (*b* = − 3.14E-03, SE = 1.01E-03, *p* = < 0.01) is shown in Fig. 1a, which splits individuals into those whose number of years in education is less than the overall national average (8.95 years, calculated as an average of the county education averages, based on census data) and those whose number of years in education is more than the overall national average. Figure 1a plots average happiness of these two groups in each county as a function of county average number of years in education showing less educated individuals are happier in counties that have higher average education, compared to less educated individuals in less educated counties. The difference in happiness for the more educated in these different counties is much smaller, though a similar trend is indicated.

**Table 3:** Model containing interaction effects between individual and county level factors predicting wellbeing and depressive symptoms (Page 10, Line 256)

	Happiness		Life Satisfaction		Depressive symptoms	
	Estimate	SE	Estimate	SE	Estimate	SE
Intercept	−10.55**	3.87	−7.20	3.94	36.83**	14.26
Years in Education	0.04***	9.14E-03	0.03***	9.27E-03	−0.29***	0.03
Employment status	−0.01	0.04	−0.14**	0.04	0.40**	0.15
Single	0.20	0.36	0.42	0.34	−0.24	1.21
Cohabitation	−1.33	2.26	−5.04*	2.33	8.78	8.36
Divorced	1.35	0.88	−0.21	0.91	0.54	3.28
Widowed	0.12	0.39	−0.21	0.41	−0.54	1.47
County average education	0.04	0.03	0.03	0.03	−0.28**	0.10
County unemployment rate	0.21	0.26	−0.48	0.27	0.52	0.96
County marriage rate	0.02***	0.43	1.12*	0.44	−1.92	1.58
Interaction: Education	−3.14E-03**^a^	1.01E-03	−3.77E-03***^a^	1.02E-03	0.03***^a^	3.66E-03
Interaction: Unemployment	0.03	0.12	0.41**^a^	0.12	−1.03*	0.44
Interaction: Single	−6.32E-03	5.13E-03	−7.81E-03	0.48	0.01	0.02
Interaction: Cohabitation	0.01	0.03	0.07*	0.03	−0.11	0.11
Interaction: Divorced	−0.03*	0.01	−3.76E-03	0.01	5.33E-03	0.05
Interaction: Widowed	−5.72E-03	5.55E-03	4.35E-04	5.76E-03	0.02	0.02
Age	−0.04***	2.48E-03	−0.02***	2.55E-03	9.06E-03	9.15E-03
Age squared	4.46E-04***	2.47E-05	3.41E-04***	2.55E-05	−2.19E-04*	9.14E-05
Female	0.10***	0.01	0.11***	0.01	0.26***	0.04
Non Han	0.04	0.03	0.01	0.03	−0.28*	0.12
Urban	0.01	0.02	−0.11***	0.02	0.02	0.07
Health	0.16***	5.96E-03	0.15***	6.15E-03	−1.26***	0.02
Log income per capita	0.15	0.08	0.08	0.08	−0.27	0.28
Log asset per capita	1.04***	0.31	0.86**	0.32	−2.12	1.15
Log county GDP per capita	0.07	0.07	−0.08	0.07	−0.15	0.27
Log county asset per capita	0.81*	0.33	0.59	0.34	−1.90	1.23
Interaction: Income	−3.53E-03	7.53E-03	5.58E-03	7.66E-03	−1.26E-03	0.03
Interaction: Asset	−0.07**	0.03	−0.05*	0.03	0.15	0.10
Observations	26,682		27,196		26,981	
N_families_	12,501		12,521		12,470	
N_counties_	158		158		158	
AIC	72,293.46		75,513.78		143,623.00	

*Note.* Multilevel model containing main effects and interactions effects between individual education level, employment status and relationship status, and county level average education, employment rate predicting marriage rate, predicting happiness, life satisfaction and depressive symptoms. Employment status was coded as 0 for being employed and 1 for being unemployed. Relationship status was added into the model as a set of dummy variables. The relationship dummy set’s reference state was being married. *p*-values were adjusted using the Benhamini-Hochberg False Discovery Rate Procedure to control for multiple testing of the effects of interest, thus we only indicate FDR significance for the interaction effects of education, employment and marriage

*p < 0.05, **p < 0.01, ***p < 0.001, ^a^FDR

Figure 1b shows the interaction effect of education on life satisfaction (*b* = − 3.77E-03, SE = 1.02E-03, *p* < 0.001). We can see opposite effects for the high and low educated groups – that those with more education have higher life satisfaction in counties with lower average education compared to their counterparts in more educated counties, while individuals with less education have higher life satisfaction in counties with higher average education compared to their counterparts in counties with lower average education. Figure 1c shows the interaction effect of education on depression (*b* = 0.03, SE = 3.66E-03, *p* < 0.001). We see that those with low education have lower depressive symptoms in counties with higher average education compared to lower educated individuals in counties with lower average education. A similar smaller effect is seen for individuals with high education. Table 3 also shows that an interaction effect also exists between individual unemployment and county unemployment rates for life satisfaction (*b* = 0.41, SE = 0.12, *p* < 0.01). Figure 1d shows this interaction effect whereby employed individuals are less satisfied with their life in counties where unemployment rates are high compared to in counties where unemployment rates are low, while effects for the unemployed are very small. Supplementary Table 6 shows the model for participants aged 18 to 59. Results were comparable, however in this model, we did not find a significant interaction effect for education on the outcomes of happiness and life satisfaction and employment for the outcome of life satisfaction, though betas were in the same direction.

### Group differences

While slight differences were seen in effect sizes from models that separated males and females (Supplementary Tables 7 and 8), results were generally comparable with those of the overall combined model. The only notable difference that emerged was the main effect of unemployment. Men who were unemployed were less happy (*b* = − 0.05, SE = 0.02, *p* < 0.01), less satisfied with life (*b* = − 0.05, SE = 0.02, *p* < 0.05) and had more depressive symptoms (*b* = 0.21, SE = 0.07, *p* < 0.01) than men who were employed, while associations between employment and mental health for women were weakly supported with unemployed women being happier (b = 0.40, SE = 0.20, *p* < 0.05).

Similarly, results were generally comparable with the overall combined model when we built models separating urban and rural dwellers (Supplementary Tables 9 and 10). One notable difference was the association between individual education level and life satisfaction, which is small and non-significant for urban dwellers (b = − 1.71E-03, SE = 2.55E-03, *p* = 0.50) and positive for rural dwellers (b = 4.70E-03, SE = 2.29E-03, *p* < 0.05). Standard deviation and range of education levels were similar in both urban and rural samples so our results were not due to a restriction of range effect.

## Discussion

This is the first study that looks at contextual effects of neighbourhood education level, employment rate and marriage rate on the outcomes of mental wellbeing and depressive symptoms in a Chinese sample. At the individual level, spending more years in education and being married is associated with better outcomes. Individuals who are less educated tend to have better outcomes in more educated counties, compared to their counterparts in less educated counties. More educated individuals tend to have lower life satisfaction in more educated counties compared to such individuals in less educated counties. Employed individuals are less satisfied with life in counties where the unemployment rate is high compared to low, while differences were minimal for the unemployed. A 1% increase in the marriage rate within a county is associated with a 0.33% increase in individual happiness and a 0.24% increase in individual life satisfaction. We discuss these results in more detail in the following sections.

### Education

At the individual level, our analysis showed that, controlling for factors such as age, gender and income, individuals who have spent more years in education also have higher levels of happiness and lower levels of depressive symptomatology. This result is in line with previous studies in China [10, 11] and worldwide [6]. However these effects are extremely small and likely not of ecological significance. Contextual education level was not associated with our outcomes, though significant interactions were found.

For our happiness outcome, we found that individuals who were less educated tended to be happier in more educated counties, with a smaller effect for more educated individuals. We also saw that higher county education predicted lower depressive symptomatology. Living in deprived neighbourhoods has been shown to be associated with lower levels of life satisfaction and higher levels of depressive symptoms/depression incidence in Western societies [17–23]. Higher neighbourhood income and local employment levels have been independently shown to be associated with decreased risk of depression [26, 27] and higher individual wellbeing [6, 28–31]. Low neighbourhood education level is another indicator of neighbourhood deprivation, and so our finding that higher contextual education is associated with more happiness and fewer depression symptoms is in line with previous findings. A more educated county may indicate greater availability of resources – in addition to better access to education the county may also have better access to healthcare (including mental health) and other public services. This may be a result of county level governing decisions. These resources may be required and accessed more often by disadvantaged individuals so having a greater county level effect on them.

Our results for life satisfaction in relation to education suggest that we are tapping into additional mechanisms here. We found that more educated individuals tend to have lower life satisfaction in more educated counties compared to such individuals in less educated counties. This effect may suggest that social status benefits [36] are reduced when higher education is more of a norm. In general, more educated individuals enjoy feelings of achievement and competence from academic successes, leading to their higher wellbeing. Achievement particularly leads to life satisfaction, and less to happiness or depression, because life satisfaction comes from an evaluation of one’s achievements and aspirations, while happiness is linked more to positive experience [54] and depressive symptomatology is linked more to affect. The definition of “achievement” however is highly contextual – gaining a university degree can be deemed a great achievement in areas where few people even go to university, while in an area where a university degree is the norm, gaining one will be less of a notable achievement. Therefore, the life satisfaction gains from academic success will vary for highly educated individuals in more and less educated counties who will evaluate their own achievement differently due to social comparison effects.

Our results showed an opposite effect for less educated people and life satisfaction – they are actually more satisfied with life when they are in a county with higher average education. We could be tapping into similar effects as seen for our happiness and depression outcomes. If social comparison effects are still at play for these individuals, this supports the view that not all forms of upward social comparison are negative [55]. Chinese people have a core belief that education, talent and hard work are key factors to economic success, and this gives them hope and belief in social mobility [56]. Therefore, in counties with higher average education, those with lower education may admire and be encouraged by the general high education around them.

We can compare our results for education with a study conducted by Nikolaev (2016) [36] with a sample of Australians. In contrast to the predominantly positive correlation between wellbeing and contextual education in our findings, the study found that higher “reference group education” was associated with lower level of life satisfaction. Furthermore, opposite to our finding, Nikolaev found people with higher education were less likely to be negatively affected by relative education. This discrepancy may be due to cultural differences or the difference in reference group used: Nikolaev’s reference group is based on age and gender, while our current reference group is based on geography.

### Employment

At the individual level, unemployed individuals were no different from employed individuals in their mental health scores though we did find more nuanced results when we broke down the unemployed group according to their different reasons for unemployment. However, the imbalance of group numbers in the different unemployment reason groups, along with ambiguous “other” and “unspecified” groups (which may be capturing all the individuals who simply cannot find a job) make us hesitant to put too much weight on these findings, though they provide a useful suggestion for future research. We found that unemployed men had lower wellbeing and higher levels of depressive symptoms than employed men, while effects were minimal for women. This sex difference is similar to previous findings from Western cultures [28, 57].

We found that employed individuals were less satisfied with life in counties where the unemployment rate was high compared to low. The life satisfaction of the unemployed did not seem to vary with county unemployment rate. This interaction effect suggests that, similar to the West, employed individuals may view high local unemployment rates as a source of worry for the security of their own jobs, they may feel sympathy towards the unemployed, or they may be affected by the repercussions of high unemployment in a region (e.g. crime) [32].

### Marriage

At the individual level, we found evidence that individuals who were single, divorced or widowed had much lower levels of happiness and life satisfaction, and much higher levels of depressive symptoms than those who were married, replicating the effect sizes of previous studies in China [7, 10, 11] and in the West [5]. Marriage brings spousal social support and other positive benefits that are beneficial for wellbeing [58, 59]. We also found evidence suggestive of a negative effect of cohabitation, compared to marriage. The family unit is considered the building block of a harmonious and stable society in China [60]. Policies also favour the married in terms of tax benefits, giving them a higher quality of life. We must remember that reverse causality may also be at play whereby people who have lower wellbeing and more depressive symptoms may be more likely to be single, divorced or even widowed.

We found that individuals were happier and more satisfied with their lives in counties where the marriage rates were higher – this effect applied to individuals of all relationship statuses. It could be that higher marriage rates indicate greater social cohesion and a more family-oriented community. In the West, marriage has been shown to stabilize interpersonal relationships and foster collective efficacy, which in turn lowers community crime [61]. It has also been associated with community wellbeing [62]. Community level marriage rate could be linked to social capital, which has been shown to mitigate against the negative mental health impacts of widowhood and living alone [63].

### Happiness, life satisfaction and depression

Our results highlight the difference between happiness, life satisfaction and depression constructs [14–16]. The difference in results that we find across our different constructs of wellbeing and depression could have emerged due to the different measures tapping into different considerations and reflections: The measure of happiness taps into emotional considerations of feelings. The measure of life satisfaction is a cognitive and self-reflective appraisal process. The measure of depression taps into the mental illness spectrum. While many studies focus on depression, less have used wellbeing measures. Understanding that differences exist between wellbeing and depression should encourage future studies to consider using both types of measures for their outcomes to give us more comprehensive interpretations.

### Limitations and future directions

Our study has several strengths including the large sample size used, the availability of census data to capture contextual effects, and inclusion of all predictors in one model to examine relative effects. However there are also several ways in which we are limited by the data available. Our measures of happiness and life satisfaction were only single item measures, which could reduce reliability. Interviews are subject to desirability bias as participants may be hesitant to disclose depressive symptoms due to stigma against mental illness. Given the small sample size, we exclude migrants but migrants would be an interesting group to analyse individually in the future. Migration is a significant social phenomenon in China and migrants have been shown to have distinctly different demographic profiles while being allowed limited access to public services in their destination cities [49]. Small rural counties are not included in the data collection, so there is no representation of extremely rural areas. The examination of the influence of contextual factors on individuals in extremely rural areas warrants its own investigation. The lifestyle of the extremely rural Chinese differ very much from the rest of China and the influence of contextual factors will differ. This will be an interesting avenue for comparison in future studies.

One major limitation is the use of 2010 census data. Certainly, there have been many changes and advances to the Chinese economy, politics and influence on the global stage in the past decade between 2010 and 2020. But using 2010 data can still give us insights. China’s GDP has been exponentially growing since the entry into the new millennium. By 2010, China was already making an impact on the global economic stage, given the 2008 financial crisis which devastated Western economies without affecting China’s stability to as much as an extent. Therefore the insights that we make from the 2010 data will still be relevant to the current times, given the similarities in economic growth between the 2000s and 2010s. Similarly, the cultural changes of the 2010s are a continuation based on the momentum gained in the 2000s. Future studies using more recent data can also use our results as longitudinal comparisons which will provide addition insight.

We had very small numbers who were single, cohabiting, divorced or widowed compared to our much larger married group – more balanced group sizes may give us better estimates (though this balance would not be representative of the real world). We assumed county as our contextual level unit and our findings provide evidence that the impact of contextual factors at the county level does exist. However, our effect sizes may be stronger using a contextual group of a smaller geographical range or with individuals within a direct social circle. A more accurate way of identifying reference groups may be to use social network information. By finding out who individuals interact with the most – perhaps family, people in the work environment or friends – we may get a better insight into who they actually compare themselves to.

We saw insightful differences in the effect of individual and contextual factors on the different measures of wellbeing and depression. A next step is to expand this research to look at the individual and contextual factors that influence a range of positive psychological traits. This is especially important because these positive psychological traits such as optimism, gratitude, meaning in life and basic psychological needs, though related, have different outcomes and correlates. The use of longitudinal data will also provide more insight into the causal pathways. We also need to look into processes that buffer against negative effects of neighbourhood factors, such as social relations and social capital [20], with the aim of developing effective interventions and public policies.

Finally, not all people behave the same. Individual differences will cause variation in how people view their external environment – some people may be more likely to socially compare themselves with others in a negative way, while others might be inspired and encouraged by people around them. Cultural differences such as those between China and Western societies may cause such differences, but individual level factors such as personality and appraisal styles may also be important. Further work can explore the intricate ways in which people socially compare themselves for better or for worse.

### Policy implications

Wellbeing itself is a new way of evaluating the expenditure of government resources. Considering individual and national wellbeing, in addition to the standard practice of using Gross Domestic Product (GDP) as the main form of utility, can help in the development of welfare, antipoverty and tax policies [32]. Based on our findings, we make some recommendations for Chinese policy. Firstly, we interestingly find that relationship status, both at the individual and contextual level, produces the strongest effects on wellbeing and depressive symptoms. This suggests that more government resources could be directed toward policies aimed at supporting unmarried individuals to combat reductions to their wellbeing. Our evidence can also give policy makers the assurance that there are no negative social comparison effects acting on wellbeing that might oppose the positive effects of such marriage policies. This also applies to employment policies. Secondly, we recommend that policy makers take closer consideration of the effects of education policies. Our evidence does suggest that an overall wellbeing benefit will be seen from increasing national education level, namely for happiness and depression levels, but such policies may overestimate the benefit to national life satisfaction because negative social comparison effects may be at play. Research now needs to look into interventions that could positively change the cognitive and emotional processes of social comparison. Social comparison can be a means of encouraging self-improvement [64, 65] and there is evidence to suggest that optimism can moderate the link between social comparison and depression [66]. Interventions could consider tapping into such moderators and comparison strategies. Such interventions, when implemented before or simultaneous to educational policies, could offset the negative impact of increasing national education on more educated individuals.

## Conclusion

Our work highlights the importance of including contextual factors in studies of wellbeing since they can have main and interaction effects that are not captured by studies looking only at individual level predictors. Our current findings suggest that national Chinese policies designed to improve employment and marriage rates will be beneficial for all but closer examination is required for educational policies. More research needs to look into interventions that encourage positive social comparison strategies (such as encouraging self-improvement or increasing optimism), so that negative social comparison effects do not offset the overall positive effect of increasing national education levels.

## Supplementary Information

**Additional file 1.**

## Data Availability

The datasets analysed during the current study are available on the Peking University Open Research Data Platform, upon registration. For more information, please refer to http://www.isss.pku.edu.cn/cfps/en/data/public/index.htm and https://opendata.pku.edu.cn/.

## Abbreviations

CFPS: China Family Panel Studies
FDR: Benjamini-Hochberg False Discovery Rate
