# Supplementary material for: Keeping up with the Wangs: individual and contextual influences on mental wellbeing and depressive symptoms in China
Source: BMC Public Health. 2022 Mar 29;22:611. doi: 10.1186/s12889-022-12869-8 (PMC8962056; doi:10.1186/s12889-022-12869-8)
Supplement: Supplementary file 1 — Additional file 1. [file 12889_2022_12869_MOESM1_ESM.docx]

Supplementary Table 1. Breakdown of employment categories

|  | N | % |
| --- | --- | --- |
| Employed | 14920 | 50% |
| *Reason for unemployment:* |  |  |
| Participating in training | 29 | 0% |
| Do not want to work | 377 | 1% |
| Have enough economic capability and hence no need to work | 241 | 1% |
| Retired | 2299 | 8% |
| Housework | 2891 | 10% |
| Too old and feeble (mainly referring to elderly farmers) | 3726 | 13% |
| No working capacity due to disability/illness | 892 | 3% |
| Other | 1737 | 6% |
| Unspecified | 2487 | 8% |

Supplementary Table 2. Breakdown of relationship status categories

|  | N | % |
| --- | --- | --- |
| Married | 24747 | 83.03% |
| Single | 2656 | 8.91% |
| Cohabitation | 66 | 0.22% |
| Divorced | 411 | 1.38% |
| Widowed | 1925 | 6.46% |

Supplementary Table 3. Correlations between individual mental health measures and individual-level predictors.

|  | Happiness | Life satisfaction | Depression |
| --- | --- | --- | --- |
| Happiness | 1 |  |  |
| Life satisfaction | 0.51*** | 1 |  |
| Depression | -0.31*** | -0.27*** | 1 |
| Education years | 0.12*** | 2.62E-03 | -0.16*** |
| *Unemployment dummy set (baseline: employed)* | | | |
| Unemployment dummy 1: Participating in training | -2.29E-03 | -4.43E-03 | 9.70E-05 |
| Unemployment dummy 2: Do not want to work | -1.58E-03 | -0.01* | -6.88E-03 |
| Unemployment dummy 3: Have enough economic capability and hence no need to work | 0.04*** | 0.04*** | -0.02*** |
| Unemployment dummy 4: Retired | 0.07*** | 0.06*** | -0.10*** |
| Unemployment dummy 5: Housework | -6.74E-03 | 0.01* | 5.12E-03 |
| Unemployment dummy 6: Too old and feeble (mainly referring to elderly farmers) | -0.01 | 0.03*** | 0.08*** |
| Unemployment dummy 7: No working capacity due to disability/illness | -0.09*** | -0.07*** | 0.17*** |
| Unemployment dummy 8: Other | -0.01* | -0.03*** | 5.90E-04 |
| *Relationship dummy set (baseline: married)* |  |  |  |
| Relationship dummy 1: Single | -0.02*** | -0.04*** | 4.81E-03 |
| Relationship dummy 2: Cohabitation | -2.99E-03 | -9.00E-03 | 6.19E-03 |
| Relationship dummy 3: Divorced | -0.07*** | -0.06*** | 0.02*** |
| Relationship dummy 4: Widowed | -0.05*** | 8.77E-03 | 0.09*** |
| Unemployed | -4.56E-03 | 9.98E-03 | 0.06*** |
| Married | 0.07*** | 0.05*** | -0.07*** |

*p<0.05, **p<0.01, ***p<0.001

Note. Unemployed is a dichotomous variable that places those who are unemployed in one group and those who are unemployed, whatever the reason, in another group. Married is a dichotomous variable that places those who are married in one group and those who are single/divorced/widowed in another group.

Supplementary Table 4. Happiness, life satisfaction and depressive symptoms predicted by individual and contextual level education, employment status and relationship status for working age sample (ages 18 to 59)

|  | Happiness | | Life Satisfaction | | Depression | |
| --- | --- | --- | --- | --- | --- | --- |
|  | Beta | SE | Beta | SE | Beta | SE |
| Intercept | 1.13* | 0.50 | 0.92 | 0.51 | 11.34*** | 1.82 |
| Years in Education | 0.01*** | 0.00 | 3.10E-03 | 0.00 | -0.03*** | 0.01 |
| Unemployed | -0.03* | 0.02 | -0.03 | 0.02 | 0.05 | 0.05 |
| Single | -0.31*** | 0.03 | -0.22*** | 0.03 | 0.77*** | 0.10 |
| Cohabitation | -0.17 | 0.13 | -0.05 | 0.13 | 0.81 | 0.48 |
| Divorced | -0.67*** | 0.05 | -0.51*** | 0.05 | 1.05*** | 0.19 |
| Widowed | -0.59*** | 0.05 | -0.29*** | 0.05 | 1.66*** | 0.17 |
| County average education | 0.02 | 0.03 | -6.80E-03 | 0.03 | -0.07 | 0.09 |
| County unemployment rate | 0.17 | 0.25 | -0.37 | 0.25 | 0.78 | 0.92 |
| County marriage rate | 0.02*** | 0.00 | 0.01** | 0.00 | -0.03 | 0.02 |
| Age | -0.06*** | 0.01 | -0.06*** | 0.01 | 0.05** | 0.02 |
| Age squared | 0.00*** | 0.00 | 7.24E-04*** | 0.00 | -7.44E-04*** | 0.00 |
| Female | 0.11*** | 0.01 | 0.12*** | 0.01 | 0.24*** | 0.05 |
| Non Han | 0.05 | 0.03 | 5.49E-03 | 0.03 | -0.17 | 0.12 |
| Urban | 0.01 | 0.02 | -0.11*** | 0.02 | 0.05 | 0.07 |
| Health | -0.17*** | 0.01 | -0.16*** | 0.01 | 1.25*** | 0.03 |
| Log income per capita | 0.11*** | 0.01 | 0.13*** | 0.01 | -0.27*** | 0.03 |
| Log asset per capita | 0.19*** | 0.03 | 0.25*** | 0.03 | -0.33** | 0.12 |
| Log county GDP per capita | 0.07* | 0.03 | -6.23E-07 | 0.03 | -0.22 | 0.12 |
| Log county asset per capita | -0.13*** | 0.04 | -0.11** | 0.04 | 0.06 | 0.13 |
| Observations | 20684 |  | 21191 |  | 21060 |  |
| Nfamilies | 10565 |  | 10594 |  | 10555 |  |
| Ncounties | 158 |  | 158 |  | 158 |  |
| AIC | 56213.7 |  | 58999.11 |  | 111095.6 |  |

*p<0.05, **p<0.01, ***p<0.001

Note. The effect of age on wellbeing has been established to be non-linear. Adding age square accounts for non-linear effects.

Blanchflower, D. G. (2021). Is happiness U-shaped everywhere? Age and subjective well-being in 145 countries. *Journal of Population Economics*, *34*(2), 575-624.

Supplementary Table 5. Happiness, Life Satisfaction and Depression predicted by individual and contextual level education, employment status (dummy variables) and relationship status (dummy variables)

|  | Happiness | | Life Satisfaction | | Depression | |
| --- | --- | --- | --- | --- | --- | --- |
|  | Beta | SE | Beta | SE | Beta | SE |
| Intercept | 0.810 | 0.46 | 0.31 | 0.47 | 13.02*** | 1.72 |
| Years in Education | 8.33E-03*** | 1.69E-03 | 2.22E-04 | 1.75E-03 | -0.03*** | 6.28E-03 |
| Unemployed: Participating in training | -0.11 | 0.18 | 0.05 | 0.18 | -0.27 | 0.65 |
| Unemployed: Do not want to work | -0.04 | 0.05 | -0.03 | 0.05 | -0.15 | 0.19 |
| Unemployed: Economically capable | 0.21*** | 0.06 | 0.28*** | 0.07 | -0.29 | 0.24 |
| Unemployed: Retired | 0.06* | 0.03 | 0.10*** | 0.03 | -0.82*** | 0.10 |
| Unemployed: Housework | 2.95E-03 | 0.02 | 0.04 | 0.02 | -0.16 | 0.08 |
| Unemployed: Too old and feeble | 0.07** | 0.02 | 0.01 | 0.02 | -0.09 | 0.09 |
| Unemployed: No working capability | -0.10** | 0.04 | -0.07 | 0.04 | 1.57*** | 0.13 |
| Unemployed: Other | -0.03 | 0.03 | -0.05 | 0.03 | 0.02 | 0.09 |
| Unmarried: Single | -0.23*** | 0.03 | -0.18*** | 0.03 | 0.75*** | 0.11 |
| Unmarried: Cohabitation | -0.27* | 0.12 | -0.12 | 0.13 | 1.17* | 0.48 |
| Unmarried: Divorced | -0.58*** | 0.05 | -0.47*** | 0.05 | 0.97*** | 0.20 |
| Unmarried: Widowed | -0.29*** | 0.03 | -0.17*** | 0.03 | 1.00*** | 0.10 |
| Age | -0.04*** | 2.58E-03 | -0.02*** | 2.67E-03 | -3.57E-04 | 9.61E-03 |
| Age squared | 4.00E-04*** | 2.60E-05 | 2.87E-04*** | 2.69E-05 | -2.40E-05 | 9.69E-05 |
| Female | 0.08*** | 0.01 | 0.08*** | 0.01 | 0.36*** | 0.04 |
| Non Han | 0.03 | 0.03 | -7.68E-03 | 0.03 | -0.17 | 0.12 |
| Urban | 0.02 | 0.02 | -0.09*** | 0.02 | 0.02 | 0.07 |
| Health | -0.16*** | 6.28E-03 | -0.15*** | 6.50E-03 | 1.18*** | 0.02 |
| Log income per capita | 0.11*** | 8.20E-03 | 0.13*** | 8.38E-03 | -0.24*** | 0.03 |
| Log asset per capita | 0.18*** | 0.03 | 0.23*** | 0.03 | -0.33** | 0.10 |
| County average education | 0.02 | 0.03 | 4.41E-03 | 0.03 | -0.09 | 0.10 |
| County unemployment rate | 0.15 | 0.25 | -0.34 | 0.26 | 0.27 | 0.95 |
| County marriage rate | 1.65*** | 0.43 | 1.25** | 0.43 | -3.35* | 1.60 |
| County log income per capita | 0.05 | 0.03 | -0.01 | 0.03 | -0.27* | 0.12 |
| County log asset per capita | -0.11** | 0.04 | -0.10** | 0.04 | 0.10 | 0.13 |
| Observations | 24838 |  | 24863 |  | 24652 |  |
| Nfamilies | 12200 |  | 12204 |  | 12153 |  |
| Ncounties | 158 |  | 158 |  | 158 |  |
| AIC | 67015.37 |  | 68773.08 |  | 131172.30 |  |

*p<0.05, **p<0.01, ***p<0.001

Supplementary Table 6. Model containing interaction effects between individual and county level education, employment and relationship status predicting happiness, life satisfaction and depressive symptoms (18-59 year olds)

|  | Happiness | | Life Satisfaction | | Depression | |
| --- | --- | --- | --- | --- | --- | --- |
|  | Beta | SE | Beta | SE | Beta | SE |
| Intercept | -8.10 | 4.45 | -6.74 | 4.53 | 18.77 | 16.01 |
| Years in Education | 0.03* | 0.01 | 5.02E-03 | 0.01 | -0.30*** | 0.04 |
| Unemployed | -0.04 | 0.05 | -0.09 | 0.05 | 0.03 | 0.17 |
| Single | 0.26 | 0.38 | 0.38 | 0.35 | -0.14 | 1.21 |
| Cohabitation | -0.23 | 2.46 | -5.39* | 2.54 | 3.52 | 8.84 |
| Divorced | 1.42 | 0.95 | -0.32 | 0.98 | -1.31 | 3.42 |
| Widowed | -2.34** | 0.81 | -0.92 | 0.84 | 0.32 | 2.90 |
| County average education | 0.03 | 0.03 | -5.16E-03 | 0.03 | -0.31** | 0.10 |
| County unemployment rate | 0.19 | 0.26 | -0.44 | 0.26 | 0.42 | 0.95 |
| County marriage rate | 0.02*** | 0.00 | 0.01** | 0.00 | -0.02 | 0.02 |
| Age | -0.06*** | 0.01 | -0.06*** | 0.01 | 0.04* | 0.02 |
| Age squared | 7.14E-04*** | 0.00 | 7.27E-04*** | 0.00 | -6.53E-04** | 0.00 |
| Female | 0.11*** | 0.01 | 0.12*** | 0.01 | 0.22*** | 0.05 |
| Non Han | 0.05 | 0.03 | 4.98E-03 | 0.03 | -0.23 | 0.12 |
| Urban | 4.12E-03 | 0.02 | -0.11*** | 0.02 | 0.05 | 0.07 |
| Health | -0.17*** | 0.01 | -0.16*** | 0.01 | 1.24*** | 0.03 |
| Log income per capita^a^ | 0.07 | 0.09 | 0.05 | 0.09 | -0.07 | 0.33 |
| Log asset per capita^a^ | 0.93** | 0.36 | 0.91* | 0.37 | -0.88 | 1.29 |
| Log county GDP per capita | 0.04 | 0.09 | -0.08 | 0.09 | 6.18E-03 | 0.31 |
| Log county asset per capita | 0.67 | 0.39 | 0.61 | 0.39 | -0.57 | 1.39 |
| Interaction Education | -1.72E-03 | 0.00 | -2.30E-04 | 0.00 | 0.03*** | 0.00 |
| Interaction unemployment | 0.03 | 0.14 | 0.19 | 0.14 | 0.19 | 0.49 |
| Interaction:Single | -8.04E-03 | 0.01 | -8.50E-03 | 0.00 | 0.01 | 0.02 |
| Interaction:Cohabitation | 7.46E-04 | 0.03 | 0.07* | 0.04 | -0.04 | 0.12 |
| Interaction:Divorced | -0.03* | 0.01 | -2.65E-03 | 0.01 | 0.03 | 0.05 |
| Interaction:Widowed | 0.02* | 0.01 | 8.88E-03 | 0.01 | 0.02 | 0.04 |
| Interaction: Income | 2.80E-03 | 0.01 | 8.65E-03 | 0.01 | -0.02 | 0.03 |
| Interaction:Asset | -0.06* | 0.03 | -0.06 | 0.03 | 0.04 | 0.11 |
| Observations | 20684 |  | 21191 |  | 21060 |  |
| N_families_ | 10565 |  | 10594 |  | 10555 |  |
| N_counties_ | 158 |  | 158 |  | 158 |  |
| AIC | 56211.26 |  | 59001.38 |  | 111063 |  |

*p<0.05, **p<0.01, ***p<0.001

^a^From census data

Supplementary Table 7. Sex differences in main effect of individual and contextual level education, employment and relationship

|  | Happiness | | | | | Life Satisfaction | | | | Depression | | | |
| --- | --- | --- | --- | --- | --- | --- | --- | --- | --- | --- | --- | --- | --- |
|  | Males | | Females | | | Males | | Females | | Males | | Females | |
|  | Beta | SE | | Beta | SE | Beta | SE | Beta | SE | Beta | SE | Beta | SE |
| Intercept | 1.27 | 0.52 | | 0.74 | 0.52 | 0.38 | 0.53 | 0.46 | 0.53 | 11.37*** | 1.77 | 14.44*** | 2.04 |
| Years in Education | 0.01*** | 2.38E-03 | | 8.17E-03*** | 2.33E-03 | 5.75E-03* | 2.43E-03 | -3.59E-03 | 2.40E-03 | -0.04*** | 8.29E-03 | -0.04*** | 9.04E-03 |
| Employment status | -0.05* | 0.02 | | 0.04* | 0.02 | -0.05* | 0.02 | 0.03 | 0.02 | 0.21** | 0.07 | -0.10 | 0.07 |
| Single | -0.43*** | 0.04 | | -0.11* | 0.04 | -0.24*** | 0.04 | -0.05 | 0.04 | 0.63*** | 0.12 | 0.80*** | 0.15 |
| Cohabitation | -0.36* | 0.16 | | -0.25 | 0.19 | -0.21 | 0.16 | -0.04 | 0.20 | 1.45* | 0.57 | 0.84 | 0.75 |
| Divorced | -0.67*** | 0.06 | | -0.61*** | 0.08 | -0.52*** | 0.07 | -0.49*** | 0.08 | 0.93*** | 0.23 | 1.13*** | 0.31 |
| Widowed | -0.21*** | 0.05 | | -0.35*** | 0.03 | -0.09* | 0.05 | -0.22*** | 0.03 | 0.96*** | 0.16 | 1.08*** | 0.13 |
| County average education | 9.72E-03 | 0.03 | | 0.03 | 0.03 | -2.90E-03 | 0.03 | 0.02 | 0.03 | -0.13 | 0.09 | -0.05 | 0.11 |
| County unemployment rate | 0.18 | 0.26 | | 0.19 | 0.27 | -0.48 | 0.27 | -0.17 | 0.27 | 0.98 | 0.87 | -0.38 | 1.06 |
| County marriage rate | 1.55*** | 0.44 | | 1.68*** | 0.46 | 1.07* | 0.45 | 1.30** | 0.45 | -3.11* | 1.47 | -2.08 | 1.79 |
| Age | -0.05*** | 3.80E-03 | | -0.04*** | 3.47E-03 | -0.02*** | 3.86E-03 | -0.02*** | 3.49E-03 | 0.02 | 0.01 | 1.39E-03 | 0.01 |
| Age squared | 5.04E-04*** | 3.74E-05 | | 4.11E-04*** | 3.46E-05 | 3.56E-04*** | 3.81E-05 | 3.13E-04*** | 3.50E-05 | -3.68E-04** | 1.30E-04 | -1.29E-04 | 1.32E-04 |
| Non Han | 0.04 | 0.04 | | 5.02E-03 | 0.04 | 0.03 | 0.04 | -0.01 | 0.04 | -0.11 | 0.15 | -0.33* | 0.15 |
| Urban | -4.10E-03 | 0.02 | | 0.02 | 0.02 | -0.13*** | 0.02 | -0.08** | 0.02 | 0.01 | 0.08 | -9.26E-03 | 0.09 |
| Health | -0.19*** | 9.06E-03 | | -0.18*** | 8.13E-03 | -0.18*** | 9.28E-03 | -0.15*** | 8.38E-03 | 1.31*** | 0.03 | 1.33*** | 0.03 |
| Log income per capita | 0.10*** | 0.01 | | 0.11*** | 9.51E-03 | 0.13*** | 0.01 | 0.13*** | 0.01 | -0.25*** | 0.04 | -0.28*** | 0.04 |
| Log asset per capita | 0.16*** | 0.04 | | 0.21*** | 0.03 | 0.25*** | 0.04 | 0.22*** | 0.04 | -0.25* | 0.12 | -0.44** | 0.13 |
| Log county GDP per capita | 0.06 | 0.03 | | 0.05 | 0.03 | -0.01 | 0.03 | -0.03 | 0.03 | -0.22* | 0.11 | -0.22 | 0.13 |
| Log county asset per capita | -0.09* | 0.04 | | -0.13** | 0.04 | -0.10* | 0.04 | -0.08* | 0.04 | 0.09 | 0.13 | -1.90E-03 | 0.15 |
| Observations | 12982 |  | | 13700 |  | 13151 |  | 14045 |  | 13066 |  | 13915 |  |
| N_families_ | 10516 |  | | 10841 |  | 10550 |  | 10882 |  | 10497 |  | 10805 |  |
| N_counties_ | 158 |  | | 158 |  | 158 |  | 158 |  | 158 |  | 158 |  |
| AIC | 35940.27 |  | | 37439.84 |  | 37112.72 |  | 39345.25 |  | 68818.22 |  | 75785.04 |  |

*p<0.05, **p<0.01, ***p<0.001

Supplementary Table 8. Sex differences in interaction effect between individual and contextual level education, employment and relationship

|  | Happiness | | | | Life Satisfaction | | | | | Depression | | | | | |  |  |
| --- | --- | --- | --- | --- | --- | --- | --- | --- | --- | --- | --- | --- | --- | --- | --- | --- | --- |
|  | Male | | Female | | | Male | | Female | | | Male | | Female | | | |  |
|  | Beta | SE | Beta | SE | | Beta | SE | Beta | SE | | Beta | SE | | Beta | SE | | |
| Intercept | -9.30* | 4.65 | -13.12** | 4.86 | | -4.55 | 4.76 | -12.62* | 5.12 | | 26.67 | 16.10 | | 35.20 | 19.38 | | |
| Years in Education | 0.05*** | 0.01 | 0.04** | 0.01 | | 0.04** | 0.01 | 0.04** | 0.01 | | -0.34*** | 0.05 | | -0.32*** | 0.05 | | |
| Employment status | -0.08 | 0.06 | 0.01 | 0.06 | | -0.14* | 0.06 | -0.15* | 0.06 | | 0.29 | 0.21 | | 0.43 | 0.23 | | |
| Single | 0.01 | 0.50 | 0.61 | 0.58 | | 0.79 | 0.48 | 0.31 | 0.51 | | -1.06 | 1.64 | | 0.88 | 1.92 | | |
| Cohabitation | -0.21 | 2.98 | -3.84 | 3.44 | | -2.23 | 3.06 | -7.47* | 3.53 | | 15.29 | 10.41 | | 11.06 | 13.25 | | |
| Divorced | 0.30 | 1.18 | 2.77* | 1.38 | | -1.53 | 1.22 | 1.56 | 1.42 | | 0.93 | 4.15 | | -0.70 | 5.32 | | |
| Widowed | 1.28 | 0.74 | -0.26 | 0.49 | | 0.59 | 0.76 | -0.47 | 0.50 | | -0.42 | 2.61 | | -0.86 | 1.88 | | |
| County average education | 0.04 | 0.03 | 0.05 | 0.03 | | 0.02 | 0.03 | 0.04 | 0.03 | | -0.40*** | 0.10 | | -0.23* | 0.11 | | |
| County unemployment rate | 0.19 | 0.27 | 0.19 | 0.30 | | -0.55 | 0.28 | -0.45 | 0.29 | | 0.77 | 0.92 | | 0.24 | 1.15 | | |
| County marriage rate | 1.50** | 0.45 | 1.61*** | 0.47 | | 1.10* | 0.46 | 1.16* | 0.46 | | -2.48 | 1.52 | | -1.29 | 1.84 | | |
| Interaction: Education | -4.39E-03** | 1.50E-03 | -3.46E-03* | 1.41E-03 | | -3.68E-03* | 1.52E-03 | -4.90E-03*** | 1.41E-03 | | 0.03*** | 5.15E-03 | | 0.03*** | 5.32E-03 | | |
| Interaction: Unemployment | 0.10 | 0.18 | 0.10 | 0.17 | | 0.25 | 0.18 | 0.55** | 0.18 | | -0.13 | 0.61 | | -1.51* | 0.66 | | |
| Interaction: Single | -0.60 | 0.71 | -1.00 | 0.83 | | -1.45* | 0.68 | -0.50 | 0.72 | | 2.34 | 2.32 | | -0.18 | 2.72 | | |
| Interaction: Cohabitation | -0.21 | 4.10 | 4.95 | 4.73 | | 2.78 | 4.22 | 10.24* | 4.86 | | -19.10 | 14.35 | | -14.10 | 18.23 | | |
| Interaction: Divorced | -1.37 | 1.66 | -4.75* | 1.93 | | 1.42 | 1.71 | -2.87 | 1.99 | | -3.25E-03 | 5.84 | | 2.50 | 7.47 | | |
| Interaction: Widowed | -2.07* | 1.04 | -0.12 | 0.68 | | -0.96 | 1.07 | 0.37 | 0.70 | | 1.85 | 3.65 | | 2.71 | 2.65 | | |
| Age | -0.05*** | 3.80E-03 | -0.04*** | 3.49E-03 | | -0.02*** | 3.87E-03 | -0.02*** | 3.51E-03 | | 0.01 | 0.01 | | -5.26E-03 | 0.01 | | |
| Age squared | 4.99E-04*** | 3.75E-05 | 4.04E-04*** | 3.48E-05 | | 3.52E-04*** | 3.82E-05 | 3.03E-04*** | 3.52E-05 | | -3.41E-04** | 1.30E-04 | | -5.45E-05 | 1.33E-04 | | |
| Non Han | 0.05 | 0.04 | 0.01 | 0.04 | | 0.04 | 0.04 | -6.48E-03 | 0.04 | | -0.18 | 0.15 | | -0.39* | 0.15 | | |
| Urban | -8.67E-03 | 0.02 | 0.02 | 0.02 | | -0.13*** | 0.02 | -0.08*** | 0.02 | | 0.02 | 0.08 | | 0.01 | 0.09 | | |
| Health | -0.18*** | 9.07E-03 | -0.18*** | 8.13E-03 | | -0.18*** | 9.29E-03 | -0.15*** | 8.38E-03 | | 1.29*** | 0.03 | | 1.32*** | 0.03 | | |
| Log income per capita | 0.14 | 0.10 | 0.13 | 0.09 | | 0.14 | 0.10 | 0.02 | 0.10 | | -0.24 | 0.33 | | -0.20 | 0.36 | | |
| Log asset per capita | 0.95* | 0.37 | 1.28** | 0.39 | | 0.61 | 0.38 | 1.32** | 0.41 | | -1.29 | 1.30 | | -2.03 | 1.56 | | |
| Log county GDP per capita | 0.08 | 0.09 | 0.06 | 0.09 | | -0.01 | 0.09 | -0.13 | 0.09 | | -0.17 | 0.31 | | -0.11 | 0.34 | | |
| Log county asset per capita | 0.75 | 0.40 | 1.01* | 0.42 | | 0.29 | 0.41 | 1.10* | 0.44 | | -1.01 | 1.38 | | -1.74 | 1.66 | | |
| Interaction: Income | -3.81E-03 | 9.54E-03 | -2.57E-03 | 9.01E-03 | | -1.16E-03 | 9.74E-03 | 0.01 | 9.55E-03 | | -9.19E-04 | 0.03 | | -6.37E-03 | 0.04 | | |
| Interaction: Asset | -0.06* | 0.03 | -0.09** | 0.03 | | -0.03 | 0.03 | -0.09** | 0.03 | | 0.08 | 0.11 | | 0.13 | 0.13 | | |
| Observations | 12982 | NA | 13700 | NA | | 13151 | NA | 14045 | NA | | 13066 | NA | | 13915 | NA | | |
| N_families_ | 10516 | NA | 10841 | NA | | 10550 | NA | 10882 | NA | | 10497 | NA | | 10805 | NA | | |
| N_counties_ | 158 | NA | 158 | NA | | 158 | NA | 158 | NA | | 158 | NA | | 158 | NA | | |
| AIC | 35934.88 | NA | 37431.35 | NA | | 37112.28 | NA | 39321.24 | NA | | 68782.95 | NA | | 75751.65 | NA | | |

*p<0.05, **p<0.01, ***p<0.001

Supplementary Table 9. Urban-rural differences in main effect of individual and contextual level education, employment and relationship

|  | Happiness | | |  | | Life Satisfaction | | | | | Depression | | | | | |  |
| --- | --- | --- | --- | --- | --- | --- | --- | --- | --- | --- | --- | --- | --- | --- | --- | --- | --- |
|  | Urban | | | Rural | | Urban | | Rural | | | Urban | | | Rural | | |  |
|  | Beta | SE | Beta | | SE | Beta | SE | | Beta | SE | | Beta | SE | | Beta | SE | |
| Intercept | 1.00 | 0.57 | 0.29 | | 0.84 | 0.09 | 0.59 | | 0.43 | 0.82 | | 10.52*** | 1.96 | | 18.28*** | 3.20 | |
| Years in Education | 2.51E-03 | 2.40E-03 | 0.01*** | | 2.28E-03 | -1.71E-03 | 2.55E-03 | | 4.70E-03* | 2.29E-03 | | -0.03** | 8.51E-03 | | -0.04*** | 8.60E-03 | |
| Employment status | -0.02 | 0.02 | 0.01 | | 0.02 | -9.28E-03 | 0.02 | | 9.52E-03 | 0.02 | | 0.03 | 0.07 | | 0.22** | 0.07 | |
| Single | -0.37*** | 0.04 | -0.17*** | | 0.04 | -0.13** | 0.04 | | -0.15*** | 0.04 | | 0.66*** | 0.14 | | 0.68*** | 0.13 | |
| Cohabitation | -0.39* | 0.16 | -0.10 | | 0.18 | -0.20 | 0.17 | | 4.90E-03 | 0.19 | | 1.55** | 0.57 | | 0.30 | 0.73 | |
| Divorced | -0.60*** | 0.06 | -0.57*** | | 0.09 | -0.49*** | 0.06 | | -0.43*** | 0.09 | | 0.95*** | 0.20 | | 0.81* | 0.34 | |
| Widowed | -0.32*** | 0.04 | -0.27*** | | 0.04 | -0.17*** | 0.04 | | -0.20*** | 0.04 | | 1.06*** | 0.14 | | 0.97*** | 0.14 | |
| County average education | 0.03 | 0.04 | -7.19E-03 | | 0.03 | 0.00 | 0.04 | | 6.63E-03 | 0.03 | | -0.02 | 0.12 | | -0.10 | 0.14 | |
| County unemployment rate | 0.29 | 0.34 | 0.26 | | 0.35 | -0.34 | 0.35 | | 1.73E-03 | 0.34 | | -0.11 | 1.17 | | -0.83 | 1.36 | |
| County marriage rate | 1.93*** | 0.55 | 1.91** | | 0.68 | 1.55** | 0.56 | | 1.20 | 0.66 | | -0.42 | 1.86 | | -6.69* | 2.67 | |
| Age | -0.05*** | 3.70E-03 | -0.03*** | | 3.36E-03 | -0.03*** | 3.89E-03 | | -0.02*** | 3.40E-03 | | 9.49E-03 | 0.01 | | 0.01 | 0.01 | |
| Age squared | 5.50E-04*** | 3.62E-05 | 3.76E-04*** | | 3.39E-05 | 4.19E-04*** | 3.82E-05 | | 2.75E-04*** | 3.42E-05 | | -3.51E-04** | 1.28E-04 | | -1.74E-04 | 1.29E-04 | |
| Female | 0.10*** | 0.02 | 0.10*** | | 0.02 | 0.14*** | 0.02 | | 0.08*** | 0.02 | | 0.20*** | 0.06 | | 0.30*** | 0.06 | |
| Non Han | 0.03 | 0.05 | 0.03 | | 0.04 | 0.05 | 0.05 | | -0.02 | 0.04 | | -0.15 | 0.18 | | -0.23 | 0.16 | |
| Health | -0.18*** | 9.43E-03 | -0.15*** | | 7.71E-03 | -0.17*** | 0.01 | | -0.14*** | 7.79E-03 | | 1.18*** | 0.03 | | 1.30*** | 0.03 | |
| Log income per capita | 0.10*** | 0.01 | 0.12*** | | 0.01 | 0.15*** | 0.01 | | 0.13*** | 0.01 | | -0.23*** | 0.04 | | -0.31** | 0.04 | |
| Log asset per capita | 0.18*** | 0.03 | 0.20** | | 0.06 | 0.23*** | 0.03 | | 0.20*** | 0.06 | | -0.26* | 0.11 | | -0.69 | 0.23 | |
| Log county GDP per capita | 0.06 | 0.04 | 0.05 | | 0.05 | -0.02 | 0.04 | | -0.04 | 0.05 | | -0.08 | 0.14 | | -0.11 | 0.18 | |
| Log county asset per capita | -0.12* | 0.05 | -0.11 | | 0.05 | -0.09 | 0.05 | | -0.07 | 0.05 | | -0.16 | 0.16 | | 0.14 | 0.21 | |
| Observations | 11801 | NA | 14881 | | NA | 12054 | NA | | 15142 | NA | | 12016 | NA | | 14965 | NA | |
| Nfamilies | 5725 | NA | 6776 | | NA | 5737 | NA | | 6784 | NA | | 5727 | NA | | 6743 | NA | |
| Ncounties | 140 | NA | 121 | | NA | 140 | NA | | 121 | NA | | 140 | NA | | 121 | NA | |
| AIC | 31227.67 | NA | 40943.08 | | NA | 33392.16 | NA | | 42080.69 | NA | | 62260.63 | NA | | 81011.72 | NA | |

*p<0.05, **p<0.01, ***p<0.001

Supplementary Table 10. Urban-rural differences in interaction effect between individual and contextual level education, employment and relationship

|  | Happiness | | | | Life Satisfaction | | | | | | Depression | | | |
| --- | --- | --- | --- | --- | --- | --- | --- | --- | --- | --- | --- | --- | --- | --- |
|  | Urban | | Rural | | Urban | | | Rural | | | Urban | | Rural | |
|  | Beta | SE | Beta | SE | | Beta | SE | | Beta | SE | Beta | SE | Beta | SE |
| Intercept | -13.63** | 4.77 | -1.77 | 8.48 | | -9.47 | 5.05 | | -14.35 | 8.36 | 36.93* | 16.92 | 2.94 | 32.08 |
| Years in Education | 0.04** | 0.01 | -4.13E-03 | 0.02 | | 0.02 | 0.01 | | 0.02 | 0.02 | -0.28*** | 0.05 | -0.17** | 0.06 |
| Employment status | 0.02 | 0.06 | -0.09 | 0.07 | | -0.06 | 0.07 | | -0.29*** | 0.07 | 0.18 | 0.22 | 0.41 | 0.25 |
| Single | -0.30 | 0.46 | 1.00 | 0.58 | | 0.28 | 0.44 | | 0.37 | 0.53 | 2.45 | 1.48 | -4.73* | 1.98 |
| Cohabitation | -7.24* | 2.85 | 6.83 | 3.61 | | -8.50** | 3.04 | | -0.06 | 3.66 | 10.31 | 10.13 | 2.50 | 13.74 |
| Divorced | 0.94 | 1.02 | 2.50 | 1.63 | | -0.39 | 1.09 | | 0.58 | 1.66 | 0.71 | 3.64 | 0.90 | 6.27 |
| Widowed | -0.75 | 0.56 | 0.64 | 0.55 | | -0.69 | 0.60 | | 7.69E-03 | 0.56 | 0.45 | 2.00 | -1.90 | 2.11 |
| County average education | 0.06 | 0.04 | -0.02 | 0.04 | | 0.02 | 0.04 | | 0.02 | 0.03 | -0.24 | 0.13 | -0.18 | 0.14 |
| County unemployment rate | 0.39 | 0.35 | 0.08 | 0.37 | | -0.38 | 0.36 | | -0.54 | 0.36 | -0.05 | 1.20 | -0.54 | 1.44 |
| County marriage rate | 1.79** | 0.55 | 2.14** | 0.69 | | 1.45* | 0.57 | | 1.16 | 0.67 | 0.23 | 1.86 | -7.55** | 2.71 |
| Interaction: Education | -4.36E-03** | 1.41E-03 | 1.94E-03 | 1.89E-03 | | -2.81E-03 | 1.47E-03 | | -1.90E-03 | 1.88E-03 | 0.03*** | 4.91E-03 | 0.01* | 7.03E-03 |
| Interaction: Unemployment | -0.11 | 0.16 | 0.32 | 0.22 | | 0.13 | 0.17 | | 1.01*** | 0.22 | -0.33 | 0.56 | -0.60 | 0.81 |
| Interaction: Single | -0.09 | 0.66 | -1.64* | 0.81 | | -0.58 | 0.63 | | -0.73 | 0.75 | -2.64 | 2.10 | 7.61** | 2.78 |
| Interaction: Cohabitation | 9.49* | 3.94 | -9.50 | 4.94 | | 11.50** | 4.20 | | 0.08 | 5.00 | -12.16 | 14.02 | -3.01 | 18.78 |
| Interaction: Divorced | -2.19 | 1.44 | -4.30 | 2.29 | | -0.15 | 1.53 | | -1.41 | 2.32 | 0.35 | 5.12 | -0.15 | 8.78 |
| Interaction: Widowed | 0.59 | 0.79 | -1.28 | 0.77 | | 0.73 | 0.84 | | -0.28 | 0.79 | 0.84 | 2.81 | 4.03 | 2.97 |
| Age | -0.05*** | 3.71E-03 | -0.03*** | 3.36E-03 | | -0.03*** | 3.91E-03 | | -0.02*** | 3.40E-03 | 3.38E-03* | 0.01 | 0.01 | 0.01 |
| Age squared | 5.43E-04*** | 3.63E-05 | 3.78E-04*** | 3.39E-05 | | 4.16E-04*** | 3.83E-05 | | 2.76E-04*** | 3.42E-05 | -2.84E-04*** | 1.28E-04 | -1.81E-04 | 1.29E-04 |
| Female | 0.11*** | 0.02 | 0.09*** | 0.02 | | 0.14*** | 0.02 | | 0.08*** | 0.02 | 0.20*** | 0.06 | 0.30*** | 0.06 |
| Non Han | 0.04 | 0.05 | 0.03 | 0.04 | | 0.05 | 0.05 | | -0.02 | 0.04 | -0.19 | 0.18 | -0.25 | 0.16 |
| Health | -0.18*** | 9.44E-03 | -0.15 | 7.71E-03 | | -0.17*** | 0.01 | | -0.14*** | 7.79E-03 | 1.17*** | 0.03 | 1.30 | 0.03 |
| Log income per capita | 0.19 | 0.12 | 0.08 | 0.12 | | 0.24 | 0.13 | | -4.71E-03 | 0.12 | -0.22 | 0.43 | -0.04 | 0.45 |
| Log asset per capita | 1.25** | 0.38 | 0.39 | 0.68 | | 0.91* | 0.41 | | 1.48* | 0.67 | -2.21 | 1.36 | 0.43 | 2.58 |
| Log county GDP per capita | 0.14 | 0.11 | 0.01 | 0.11 | | 0.06 | 0.12 | | -0.16 | 0.11 | -0.04 | 0.38 | 0.13 | 0.44 |
| Log county asset per capita | 1.00 | 0.40 | 0.11 | 0.77 | | 0.62 | 0.43 | | 1.38* | 0.76 | -2.22 | 1.43 | 1.41 | 2.93 |
| Interaction: Income | -9.29E-03 | 0.01 | 4.25E-03 | 0.01 | | -9.32E-03 | 0.01 | | 0.01 | 0.01 | -4.19E-04 | 0.04 | -0.03 | 0.05 |
| Interaction: Asset | -0.09** | 0.03 | -0.02 | 0.06 | | -0.06 | 0.03 | | -0.11 | 0.06 | 0.16 | 0.11 | -0.10 | 0.23 |
| Observations | 11801 | NA | 14881 | NA | | 12054 | NA | | 15142 | NA | 12016 | NA | 14965 | NA |
| Nfamilies | 5725 | NA | 6776 | NA | | 5737 | NA | | 6784 | NA | 5727 | NA | 6743 | NA |
| Ncounties | 140 | NA | 121 | NA | | 140 | NA | | 121 | NA | 140 | NA | 121 | NA |
| AIC | 31214.55 | NA | 40942.47 | NA | | 33389.92 | NA | | 42067.25 | NA | 62236.96 | NA | 81013.06 | NA |

*p<0.05, **p<0.01, ***p<0.001
